# Locomotor Pattern and Force Generation Modulated by Ionic Channels: A Computational Study of Spinal Networks Underlying Locomotion

**DOI:** 10.3389/fncom.2022.809599

**Published:** 2022-04-14

**Authors:** Qiang Zhang, Yi Cheng, Mei Zhou, Yue Dai

**Affiliations:** ^1^Shanghai Key Laboratory of Multidimensional Information Processing, School of Communication and Electronic Engineering, East China Normal University, Shanghai, China; ^2^School of Physical Education, Yunnan University, Kunming, China; ^3^Key Laboratory of Adolescent Health Assessment and Exercise Intervention of Ministry of Education, School of Physical Education and Health Care, East China Normal University, Shanghai, China

**Keywords:** central pattern generator (CPG) model, locomotion, motoneuron recruitment, ionic channel, motor control

## Abstract

Locomotion is a fundamental movement in vertebrates produced by spinal networks known as central pattern generators (CPG). During fictive locomotion cat lumbar motoneurons (MNs) exhibit changes in membrane properties, including hyperpolarization of voltage threshold, reduction of afterhyperpolarization and input resistance, and amplification of nonlinear membrane properties. Both modeling and electrophysiological studies suggest that these changes can be produced by upregulating voltage-gated sodium channel (VGSC), persistent sodium (NaP), or L-type calcium channel (LTCC) or downregulating delayed-rectifier potassium (K(DR)) or calcium-dependent potassium channel (KCa) in spinal MNs. Further studies implicate that these channel modulations increase motor output and facilitate MN recruitment. However, it remains unknown how the channel modulation of CPG networks or MN pools affects the rhythmic generation of locomotion and force production of skeletal muscle during locomotion. In order to investigate this issue, we built a two-level CPG model composed of excitatory interneuron pools (Exc-INs), coupled reciprocally with inhibitory interneuron pools (Inh-INs), and projected to the flexor-extensor MN pools innervating skeletal muscles. Each pool consisted of 100 neurons with membrane properties based on cat spinal neurons. VGSC, K(DR), NaP, KCa, LTCC, and H-current channels were included in the model. Simulation results showed that (1) upregulating VGSC, NaP, or LTCC or downregulating KCa in MNs increased discharge rate and recruitment of MNs, thus facilitating locomotor pattern formation, increased amplitude of electroneurogram (ENG) bursting, and enhanced force generation of skeletal muscles. (2) The same channel modulation in Exc-INs increased the firing frequency of the Exc-INs, facilitated rhythmic generation, and increased flexor-extensor durations of step cycles. (3) Contrarily, downregulation of NaP or LTCC in MNs or Exc-INs or both CPG (Exc-INs and Inh-INs) and MNs disrupted locomotor pattern and reduced or even blocked the ENG bursting of MNs and force generation of skeletal muscles. (4) Pharmacological experiments showed that bath application of 25 μM nimodipine or 2 μM riluzole completely blocked fictive locomotion in isolated rat spinal cord, consistent with simulation results. We concluded that upregulation of VGSC, NaP, or LTCC or downregulation of KCa facilitated rhythmic generation and force production during walking, with NaP and LTCC playing an essential role.

## Introduction

Locomotion in mammals is generated by spinal cord networks known as central pattern generators (CPG) and is characterized as a periodical repetition of a simple motor pattern of limbs (Brown, 1914; Gordon and Whelan, 2006; Kiehn, 2011, 2016; Sillar et al., 2014). The CPG plays an essential role in the rhythmic generation, pattern formation, and movement coordination during locomotion. It was reported previously that during fictive locomotion, the active membrane properties of spinal motoneurons (MNs) significantly changed, including hyperpolarization of voltage threshold (Krawitz et al., 2001; Power et al., 2010; MacDonell et al., 2015), reduction of afterhyperpolarization (AHP, Brownstone et al., 1992) and input resistance (Shefchyk and Jordan, 1985; Gosgnach et al., 2000), and enhancement of non-linear membrane property (Brownstone et al., 1994; Powers and Heckman, 2017, 2015; Binder et al., 2020). Modeling results suggest that upregulation of voltage-gated sodium channel (VGSC) conductance and/or downregulation of delayed-rectifier potassium (K(DR)) conductance in spinal MNs are mechanisms underlying hyperpolarization of voltage threshold during fictive locomotion (Dai et al., 2002). These modeling predictions were verified later by pharmacological experiments (Power et al., 2012; Lombardo and Harrington, 2016; Dai et al., 2018). Furthermore, recent modeling studies suggest that the locomotion-induced changes in membrane properties enhance the motoneuronal excitability, upregulate motoneuronal frequency-current relationships (Dai et al., 2018), and facilitate recruitment of MN pools (Bawa et al., 2014; Zhang and Dai, 2020). The enhancement of MN recruitment could be produced by upregulating VGSC, persistent sodium (NaP), and/or L-type calcium channel (LTCC) conductance or downregulating K(DR) and/or calcium-dependent potassium channel (KCa) conductance in spinal MNs. However, it remains unclear how the above channel modulation of CPG networks could affect the generation of locomotion, the formation of locomotor patterns, and the force generation of skeletal muscles innervated by the spinal MNs during locomotion. Modulation of CPG output essentially depends on the structure of CPG networks, the synaptic connection of CPG components, and intrinsic membrane properties of CPG neurons. Since the half-center model was first proposed by Graham Brown in the 1910s (Brown, 1914), the CPG networks have been studied intensively through electrophysiological and modeling approaches. The reciprocal organization of pathways transmitting excitatory action to alpha MNes of flexors and extensors was first verified through an intracellular recording from the cat spinal cord with DOPA (Jankowska et al., 1967a,b). Subsequent work further revealed the details of CPG networks underlying locomotion in mammals, including the alternation of flexors and extensors induced by noradrenergic precursor levodopa (L-DOPA) (Grillner and Zangger, 1979), involvement of spinal interneurons (INs) of CPG networks in flexion reflexes, sensory modulation of locomotion from afferent stimulation (Guertin et al., 1995), CPG networks specifically responsible for spontaneous deletions during fictive locomotion (Lafreniere-Roula and McCrea, 2005), and multiple levels of CPG networks proposed for rhythmic generation and pattern formation (Quevedo et al., 2005; Rybak et al., 2006a,b; McCrea and Rybak, 2007, 2008). These studies briefly outline some of the important work that reveals details of spinal cord circuits for the generation of locomotion and demonstrates multiple levels of CPG networks developed for interpretation of the diversity of locomotor activities over the past many decades. Following the exploration of CPG networks, studies of channel mechanisms responsible for locomotion have been also conducted intensively in the mammalian spinal cord. Two major channels, the LTCC (Büschges et al., 2000) and NaP channels (Zhong et al., 2007; Tazerart et al., 2008; Brocard et al., 2010), have been shown to play essential roles in generating locomotion. However, contributions of channel modulation to the generation of locomotion are still a crucial issue needed to be further explored. In this study, we built a large-scale two-level CPG model capable of generating locomotor rhythm and patterns to investigate the effect of channel modulation of CPG networks and spinal MN pools on the generation of locomotion, formation of the locomotor pattern, and force production of skeletal muscles during fictive locomotion. Our study implicates that upregulation of VGSC, NaP, or LTCC or downregulation of KCa facilitated the rhythmic generation of locomotion and force production of skeletal muscle in walking.

## Materials and Methods

### Rat Experiments

Experiments were carried out in accordance with the East China Normal University Laboratory Animal Center, and all procedures were in accordance with protocols approved by the Animal Experiment Ethics Committee (Ethics No. R20201202). Experiments were performed on Sprague-Dawley rats aged 1–4 days. Animals were euthanatized by decapitation, and their spinal cords were isolated by ventral laminectomy under ice-cold (4°C), oxygenated (95% O2-5% CO2) dissecting artificial cerebral spinal fluid (aCSF). The isolated spinal cord from segments T13 to S3 was removed and pinned ventral side-up and superfused with oxygenated recording aCSF. Experiments were performed after incubating preparations in oxygenated regular recording aCSF at room temperature (20–23°C) for 30 min. Fictive locomotion was evoked by perfusion with recording aCSF containing a combination of 5-hydroxytryptamine (5-HT; 10-15 μM) and N-methyl-D-aspartate (NMDA; 3-5 μM). A small-diameter suction electrode was placed on the L2 or L5 root. Ventral root recordings were band-pass-filtered (100 to 5 kHz) and recorded using a MultiClamp 700B (Molecular Devices, Silicon Valley, CA, United States).

To make whole-cell recordings from the intact cord, a patch electrode was lowered into a small slit in the ventrolateral surface of the L2 or L5 segment. The pipette electrodes were pulled from borosilicate glass (1B150F-4, World Precision Instruments, Florida, USA) with an electrode puller (P-1000, Sutter Instrument, Novato, CA, United States) and had resistances of 6–8 MΩ when filled with intracellular solution. In this study, the control condition refers to after the 5-HT and NMDA application to the recording aCSF. A MultiClamp 700B, a Digidata 1550, a Mini-Digi 1B, and pCLAMP 10.7 (all from Molecular Devices, Silicon Valley, CA, United States) were used for data acquisition. Data were low-pass filtered at 3 kHz and sampled at 10 kHz. Electrophysiological data were analyzed with Axon Clampfit 10.7 (Molecular Devices, Silicon Valley, CA, United States). Data are presented as means ± *SD*. A paired *t*-test was used to analyze the effect of drugs on neuronal excitability using (GraphPad, San Diego, CA, United States), *P* < 0.05 for significant tests.

*Dissecting aCSF*: 25 mM NaCl, 253 mM sucrose, 1.9 mM KCl, 1.2 mM NaH2PO4, 10 mM MgSO4, 26 mM NaHCO3, 25 mM glucose, and 1 mM CaCl2.

*Recording aCSF*: 125 mM NaCl, 2.5 mM KCl, 26 mM NaHCO3, 1.25 mM NaH2PO4, 25 mM C6H12O6, 1 mM MgCl2, and 2 mM CaCl2.

*Intracellular solution*: 135 mM C5H12O7K, 10 mM NaCl, 10 mM C8H18N2O4S, 2 mM MgCl2, 5 mM Mg-ATP, and.5 mM GTP.

*Drugs*: C10H12N2O (H9523, Sigma-Aldrich, MO, United States), C5H9NO4 (M3263 Sigma-Aldrich, MO, United States), C8H5F3N2OS (HY-B0211, MCE, NJ, United States), and C21H26N2O7 (HY-B0265 MCE, NJ, United States).

### Modeling

**FIGURE 1:**
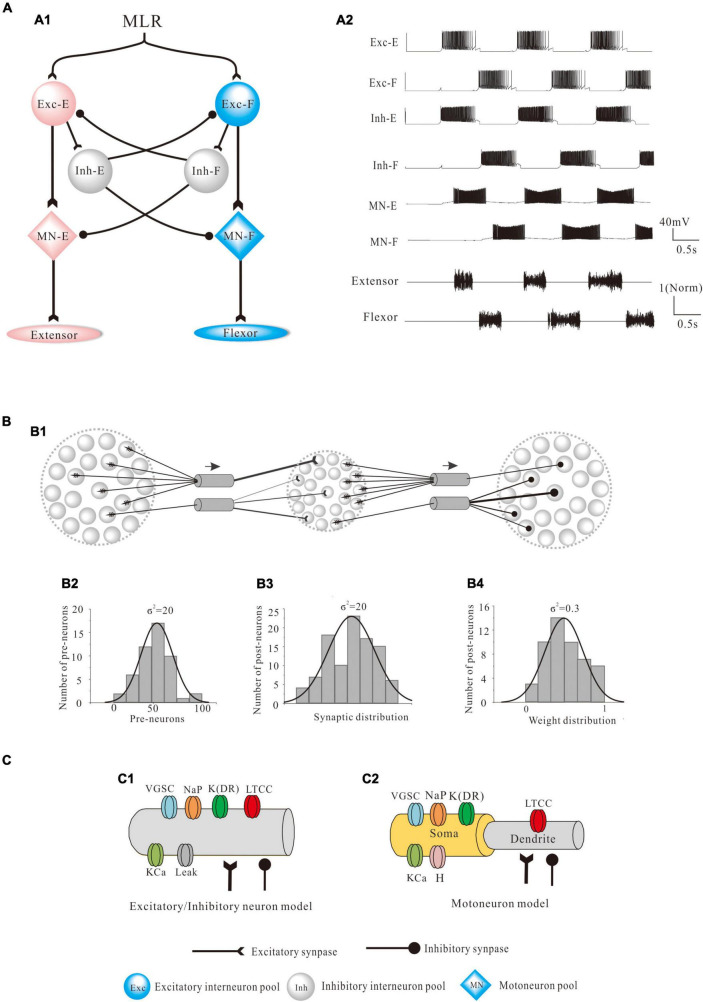
Modeling of the central pattern generator (CPG). **(A)**: The two-level structure of the CPG model is composed of excitatory interneuron pools, coupled reciprocally with inhibitory interneuron pools, and projected to the flexor-extensor motoneuron (MN) pools which innervated skeletal muscles. The two excitatory neurons pools with flexor (Exc-F) and extensor (Exc-E) represented a rhythm generator and reciprocally inhibited each other by two pools of inhibitory interneurons (Inh-E and Inh-F). The MN pools received excitatory input of Exc-E/Exc-F pools and inhibitory input of Inh-E/Inh-F pools. Each pool consisted of 100 neurons with intrinsic properties based on cat spinal neurons **(A1)**. The rhythmic activities of locomotion were generated by the CPG network from the flexor and extensor half-centers to MN pools and then to the skeletal muscles **(A2)**. The bursting of neurons was selected from the representative neurons of the pools to show the rhythmic activities of the whole spinal circuits during locomotion. **(B)**: The synaptic connections and weights based on the network **(A1)** followed Gaussian distribution **(B1)**. Specifically, the presynaptic neurons (σ^2^ = 20, **B2**), postsynaptic connections (σ^2^ = 20, **B3**), and the synaptic weights for the connections (σ^2^ = 0.3, **B4**) followed the Gaussian distribution. **(C)**: Each excitatory and inhibitory neuron was composed of a single compartment, which contained voltage-gated sodium channel (VGSC), delayed-rectifier potassium (K(DR)), persistent sodium (NaP), calcium-dependent potassium channel (KCa), and L-type calcium channel (LTCC) **(C1)**. Each MN had soma and dendrite compartments and included VGSC, K(DR), NaP, KCa, and H channels in soma and LTCC in the dendrite **(C2)**.

A CPG network model was built with NEURON 7.7 (NEURON, Yale University, New Haven, CT, United States). The large-scale two-level CPG model consisted of generators of locomotor rhythm and gait pattern. The rhythmic generator included two pools of excitatory INs representing flexor (Exc-F) and extensor (Exc-E) half-centers. The excitatory pools were reciprocally coupled by inhibitory IN pools (Inh-E and Inh-F). The MN pools consisted of 100 MNs in each pool, received synaptic inputs from excitatory and inhibitory IN pools, and innervated the flexor and extensor skeletal muscles (Figure 1A1). Figure 1A2 showed the output of the whole spinal network with a brief excitatory input delivered simultaneously to the Exc-E and Exc-F pools from the MLR. The bursting of neurons that were selected from the representative neurons of the pools showed the rhythmic activities of the spinal cord networks from the CPG to MNs and skeletal muscles. As shown in Figure 1A, the excitatory IN pools (Exc-E and Exc-F) received excitatory inputs of 500 ms from descending supraspinal tract of MLR at beginning of the simulation, and then the excitatory synaptic inputs from Exc-E and Exc-F pools were transmitted to the inhibitory IN pools (Inh-E and Inh-F) as well as the MN pools (MN-E and MN-F) simultaneously (Figure 1A1). The rhythmic bursting of the networks was mainly generated by the reciprocal inhibition of Inh-E and Inh-F pools between the extensor and flexor half-centers (Figure 1A1), and the output of the networks MN recruitment and bursting frequency) were generated by the MN pools, where the spatial and temporal summation of excitatory and inhibitory inputs were achieved to produce the network output. Finally, the number of recruited MNs was converted into the electroneurogram (ENG) bursting of the extensor and flexor (Figure 1A2), and the ENG bursting was further transformed for force generation of the skeletal muscles. Figure 1B1 illustrated the principle of network connections between the excitatory and inhibitory IN pools. Gaussian distributions were applied to the selection of presynaptic neurons (Figure 1B2), synaptic distributions to the postsynaptic neurons (Figure 1B3), and synaptic weights for the network connections (Figure 1B4). The present two-level CPG model built with the minimum network of reciprocal inhibition could generate multiple behaviors of the CPG for locomotion. Each excitatory and inhibitory pool consisted of 100 INs, and there was no synaptic connection between neurons within the same pool. Neurons in each pool were divided into 5 sub-types with different membrane properties based on cat spinal MNs and INs (Tables 1, 2). In order to reduce computing cost without losing simulating accuracy, we simplified the morphology of the excitatory and inhibitory INs as a single-compartment with conductances described by Hodgkin-Huxley equations (Tables 3, 4). Each excitatory or inhibitory IN included the VGSC, NaP, K(DR), KCa, low-voltage activated LTCC, and potassium-mediated leak channel (Figure 1C1). The MN model had soma and dendritic compartments with VGSC, K(DR), NaP, KCa, and H-current in soma and LTCC in the dendrite (Figure 1C2). The membrane properties of the model MNs and INs were based on cat lumbar MNs (Dai et al., 2002; Dai et al., 2018) and INs (Rybak et al., 2003), which included input resistance (R_in_), rheobase, afterhyperpolarization (AHP) duration, and depth, action potential (AP) height, voltage threshold (V_th_), and resting membrane potential (E_m_) (Tables 1, 2).

**TABLE 1:** Membrane properties of excitatory and inhibitory interneuron models.

Properties	Neuron 1	Neuron 2	Neuron 3	Neuron 4	Neuron 5
R_in_ (MΩ)	0.5	0.7	0.65	0.6	0.75
Rheobase (nA)	20.6	18.0	19.2	20.0	17.5
AHP duration(ms)	71.4	65.5	68.7	56.7	60.2
AHP depth (mV)	3.8	4.3	5.6	3.3	4.5
AP height (mV)	54.2	51.5	55.0	56.3	54.7
V_th_ (mV)	–48.5	–49.0	–48.4	–50.8	–50.0
Resting E_m_ (mV)	–70.1	–71.2	–70.6	–70.8	–71.8

**TABLE 2:** Membrane properties of motoneuron models.

Properties	Neuron 1	Neuron 2	Neuron 3	Neuron 4	Neuron 5
R_in_(MΩ)	0.45	0.47	0.64	0.53	0.70
Rheobase(nA)	13.3	16	10.6	12.8	18.2
AHP duration(ms)	69.6	107.3	56.5	60.2	75
AHP depth(mV)	3.2	4.2	4.4	3.0	3.7
AP height(mV)	61.0	68.6	68.5	60.0	66.8
V_th_(mV)	–52.3	–52.3	–51.6	–50.3	–53.7
Resting E_m_ (mV)	–69.9	–70.2	–71.5	–71.0	–72.0

**TABLE 3:** Morphological parameters of models.

Model		Length (μm)	Diameter (μm)	R_M_(Ω*cm2*)	R_A_(Ω*cm*)	C_M_ (μF/cm^2^)
Exc and Inh		100∼200	5∼15	7000∼10000	60∼100	1
MN	Soma	100∼300	15∼25	7000∼10000	20∼40	1
	Dendrite	400∼800	10∼15	7000∼10000	60∼80	1

**TABLE 4:** Distribution and density of ionic conductance of models (mS/cm^2^).

Model		VGSC	NaP	K(*DR*)	KCa	LTCC	Leak
Exc and Inh		100∼150	1∼3	100∼150	1∼5	0.2∼0.5	0.1∼0.5
MN	Soma	100∼300	2∼5	100∼200	2∼10	/	0.1
	Dendrite	/	/	/	/	3	10

The membrane potential in each compartment of the models was calculated based on the cable equation:


(1) $$C_{m}\frac{dV_{m}}{dt}=-\sum I_{ionic}-\sum I_{syn}$$

Where C_m_, V_m_, Ionic, and I_*syn*_ represented membrane capacitance, ionic channel currents, and synaptic currents, respectively. Ionic conductances include the following:


(2) $$\begin{array}{c} \begin{array}{c} I_{VGSC}=g_{VGSC}m^{3}h\left(V_{m}-E_{Na}\right)\\ I_{NaP}=g_{NaP}m_{NaP}s_{NaP}\left(V_{m}-E_{Na}\right)\\ I_{K\left(DR\right)}=g_{K\left(DR\right)}n^{4}\left(V_{m}-E_{K}\right) \end{array}\\ I_{KCa}=g_{KCa}n_{KCa}\left(V_{m}-E_{K}\right)\\ \begin{array}{c} I_{LTCC}=g_{LTCC}m_{LTCC}\left(V_{m}-E_{Ca}\right)\\ I_{H}=g_{H}m_{H}\left(V_{m}-E_{H}\right) \end{array}\\ I_{Leak}=g_{Leak}\left(V_{m}-E_{K}\right) \end{array}$$

Where g_VGSC_, g_NaP_, g_K(DR)_, g_KCa_, g_LTCC,_ g_H,_ and g_Leak_ were maximum conductance for VGSC, NaP, K(DR), KCa, LTCC, H-current, and leak channel, respectively. E_*Na*_, E_K_, E_Ca_, and E_H_ were reversal potentials for Na^+^, K^+^, Ca^2+^, and H-current and were set to 55, −75, 80, and −55 mV, respectively. The leak current is generally mediated by non-voltage-gated chloride and/or potassium channels. In this study, E_Leak_ was set to −75 mV (Dai et al., 2002, 2018). The resting membrane potential E_m_ of the model cells was set to −70 mV. State variables *m*, *h*, *n*, and *s* (with or without subscripts) were defined by the Hodgkin-Huxley equation (Hodgkin and Huxley, 1952a,b) as below:


(3) $$\frac{dX}{dt}=\alpha \left(1-X\right)-\beta X$$

Where steady-state value *X*_∞_ = α/(α + β)and time constant τ = 1/(α + β).

The intracellular calcium concentration ([Ca^2+^]_in_) in the compartment satisfies the following equation:


(4) $$\frac{d{\left[Ca^{2+}\right]}_{in}}{dt}=B\cdot I_{CaN}-\frac{{\left[Ca^{2+}\right]}_{in}}{\tau_{Ca}}$$

Where B is a scaling constant set to 10. _τ_Ca__ is a time constant, the rate of decay of [Ca^2+^]_in_ and is set to 15 ms. I_CaN_ is the N-type Ca^2+^ current.

The excitatory and inhibitory synaptic currents were defined by the following equations:


(5) $$\begin{array}{c} I_{exc}\left(t\right)=w_{i}g_{exc}\cdot \left(e^{-\left(t-t_{0}\right)/\tau_{1}}-e^{-\left(t-t_{0}\right)/\tau_{2}}\right)\cdot \left(V_{m}-E_{exc}\right)\\ \;\left(i=1,2,\ldots ,n\right)\\ I_{inh}\left(t\right)=w_{j}g_{inh}\cdot \left(e^{-\left(t-t_{0}\right)/\tau_{1}}-e^{-\left(t-t_{0}\right)/\tau_{2}}\right)\cdot \left(V_{m}-E_{inh}\right)\\ \;\left(j=1,2,\ldots ,n\right) \end{array}$$

Where *g*_*exc*_ represented the maximum conductance of excitatory synaptic currents (*I*_*exc*_), *g*_*inh*_ represented the maximum conductance of inhibitory synaptic currents (*I*_*inh*_). *E*_*exc*_ and *E*_*inh*_ were reversal potentials for excitatory and inhibitory synapses and set to 0 and −80 mV, respectively (Coombs et al., 1955). *w*_*i*_ and *w*_*j*_ represented the weight of synaptic inputs and followed the normal distribution. _τ_1__ and _τ_2__ were time constants for synaptic currents and set to 3 ms and 2 ms, respectively (Rybak et al., 2006a).

**FIGURE 2:**
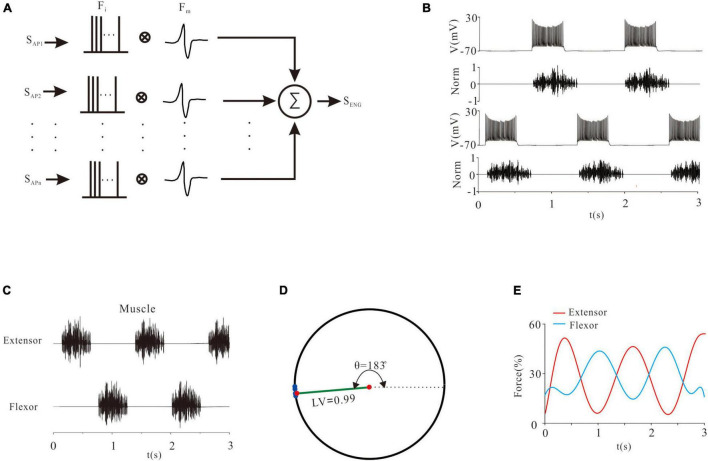
The relationship between electroneurogram (ENG) and neuronal excitability. **(A)**: A mathematical method of calculating ENG signals. The repetitive firings of each neuron were transformed into a pulse sequence (F_i_) and convolved with the wavelet signal (F_m_). Then, all the convolved signals were superimposed to form an ENG signal. **(B)**: An example of the relationship between a rhythmic discharge and ENG output. **(C)**: ENG activities of MN pools. **(D)**: A quantitative description of the locomotor pattern by the ENG signals. Blue points on the unit circle represented the phase difference (θ) between the flexor and extensor. The length of the green line represented the locomotor vector (LV). **(E)**: Force generation in flexor and extensor muscles.

Electroneurogram is an essential measurement of spinal MN output and index of CPG network for generation of locomotion. The basic component of ENG was the motor unit action potential (MUAP). The ENG signal was calculated as shown in Figure 2A. Specifically, all the time-dependent action potentials (APs) of MN pools (S_AP_) were converted to impulses (F_i_). The F_i_ and MUAP (F_M_) were convolved to the ENG signal (S_ENG_). The mathematical formula used in this study followed previous work (Moraud et al., 2016) as below:


(6) $$S_{ENG}=\sum_{t>0}\sum_{1\leq i\leq n}F_{i}⊗F_{M}$$

Where *n* was the number of neurons in a pool (*n* = 100), and *t* was the sampling time. Figure 2B showed an example of generating an ENG signal, which reflected the activity of the neural network.

We also made a quantitative description of locomotor activities. The starting time of each excitation and inhibition of flexor and extensor was recorded as *T*_*k, i*_ (*k* = 1, 2, *j* = 1, 2…, N), where *k* was the flexor or extensor, and *i* was the number of times recorded. Taking the flexor signal (or extensor signal) as a reference, the proportion of the starting time difference of the extensor signal (or flexor signal) was calculated by the formula below (Henryk et al., 2020):


(7) $$ratio_{i}=\frac{T_{2,i}-T_{1,i}}{T_{1,i+1}-T_{1,i}},i=1,2,\ldots ,N$$

The normalized data in a unit circle represented the locomotor phase. The mean phase was calculated to define the locomotor vector (LV). θ was the phase difference between the flexor and extensor (Figures 2C,D).

In order to quantify the relationship between ENG recording of MN pools and force generation of MN-innervated skeletal muscles, several approaches have been developed (Teka et al., 2017; Dideriksen et al., 2019). In this study the following mathematical equation was used:


(8) $$F=a_{0}+a_{1}f^{1}+\ldots +a_{n}f^{n}\begin{array}{cc} & \left(n=1,2,3,\ldots \right) \end{array}$$

Where *F* was the force, *f* was the instantaneous frequency of ENG, and *a*_*i*_ was the coefficient. Figure 2E showed the force generation of ENG.

## Results

### Locomotor Activity Modulated by VGSC of Motoneurons

**FIGURE 3:**
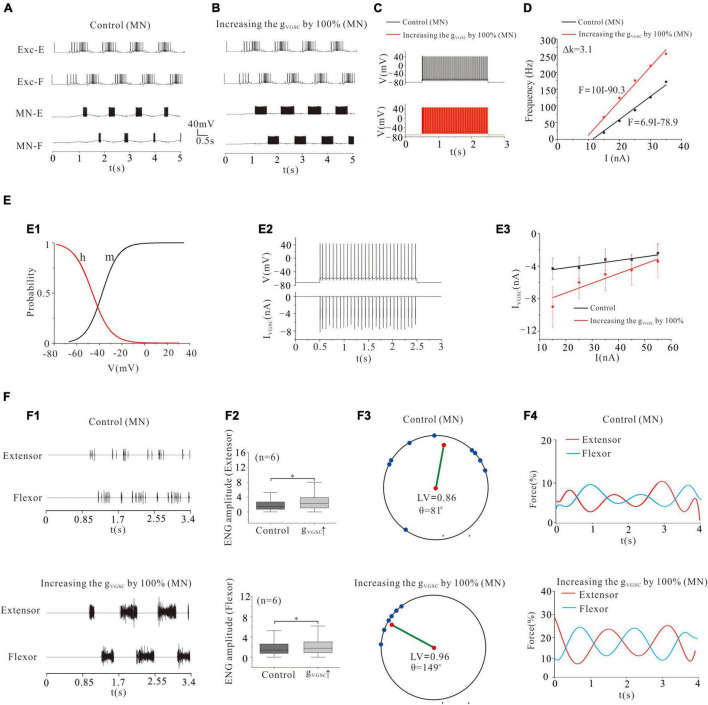
Locomotion regulated by VGSC of MNs. **(A,B)**: Increasing g_VGSC_ by 100% enhanced the rhythmic bursting. **(C)**: Repetitive firing was evoked by injecting a step current of 2-s duration and 16-nA into one of the selected MNs. Increasing g_VGSC_ by 100% increased the firing frequency of the MN from 33 Hz (control) to 90 Hz and gain of the frequency-current (F-I) relationship (**D**, ΔK = 3.1 Hz/nA). **(E)**: The activation curve (m) and the inactivation curve (h) of VGSC **(E1)** and the relationship between repetitive firing and I_VGSC_ **(E2)**. The firing was evoked by injecting a 15 nA step current into the soma. The amount of VGSC currents decreased with increasing the injected currents in both control and increase of g_VGSC_ **(E3)**. **(F)**: A 100% increase of g_VGSC_ enhanced the ENG bursting and increased ENG amplitude of extensor and flexor MN pools by 20 and 21% (*P* < 0.05, **F1,F2**), respectively. The locomotor vector (LV) increased from 0.86 (control) to 0.96 **(F3)** and force generation from 7% (control) to 16% in both extensor and flexor muscles **(F4)**. *P < 0.05.

Voltage-gated sodium channels are one of the most important ionic channels for generating APs. Previous electrophysiological experiments and modeling studies reported that cat lumbar MNs displayed V_th_ hyperpolarization and thus increased the motoneuronal excitability and recruitment through upregulation of g_VGSC_ during fictive locomotion or fictive scratching (Krawitz et al., 2001; Dai et al., 2002, 2018; Power et al., 2010, 2012; Zhang and Dai, 2020). In this study, we demonstrated that modulation of VGSC not only determined the MN recruitment but also strongly shaped the locomotor patterns and force generation of flexor and extensor activity. Increasing g_VGSC_ by 100% in MN pools facilitated the rhythmic generation of locomotion (Figures 3A,B), and enhanced the frequency of repetitive firing from 33 Hz (control) to 90 Hz (Figure 3C) and MN output of the F-I relationships (Figure 3D, ΔK = 3.1 Hz/nA). Figure 3E illustrated kinetics of VGSC with activation (m) and inactivation curves (h) (E1). The relationship between repetitive firing and peak VGSC currents is shown in Figure 3E2. The amount of VGSC currents decreased with the increase of injected currents in both control and g_VGSC_ increment (E3). The increased recruitment of MN pools further facilitated locomotor activities (Figure 3F1), increased ENG amplitude of extensor and flexor skeletal muscles by 20-21% (Figure 3F2, *P* < 0.05, *n* = 6), facilitated the formation of locomotor patterns (Figure 3F3: control: LV = 0.86, θ = 81°; VGSC: LV = 0.96, θ = 149°) and enhanced force generation by the extensor and flexor skeletal muscles (Figure 3F4: control: force = 7%, VGSC: force = 16%). These results suggested that upregulation of VGSC increased recruitment of MN pools thus enhancing the formation of locomotor patterns and force production of skeletal muscles.

### Locomotor Activity Modulated by KCa of Motoneurons

**FIGURE 4:**
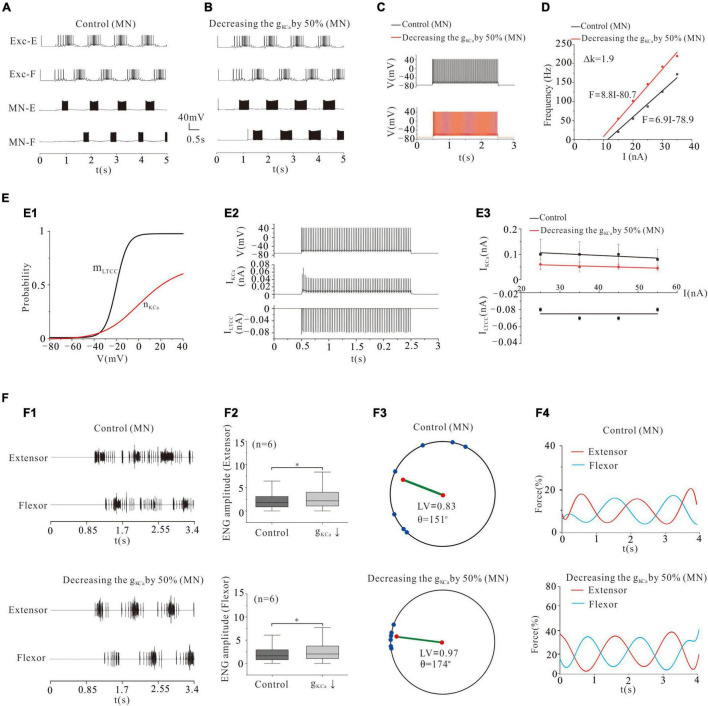
Locomotion regulated by KCa of MNs. **(A,B)**: Decreasing g_KCa_ by 50% enhanced the rhythmic output. **(C)**: Repetitive firing was evoked by injecting a step current of 2-s duration and 16-nA into one of the selected MNs. Decreasing g_KCa_ by 50% increased the firing frequency of the MN from 33 Hz (control) to 55 Hz and gain of the F-I relationship (**D**, ΔK = 1.9 Hz/nA). **(E)**: The dynamics of the activation curve of KCa (m_LTCC_ and n_KCa_, **E1**) and the relationship between repetitive firing and currents of KCa and LTCC **(E2)**. The firing was evoked by injecting a 16 nA step current into the soma. Increasing the injected current slightly decreased the amount of KCa currents in both control and g_ KCa_ decrement, and LTCC currents were almost unchanged **(E3)**. **(F)**: A 50% reduction of g_ KCa_ enhanced the ENG amplitude of extensor and flexor MN pools by 28 and 26% (*P* < 0.05, **F1,F2**), respectively. The enhancement of alternating rhythm further facilitated the locomotor pattern (**F3**, control: LV = 0.83, θ = 151°; KCa: LV = 0.97, θ = 174°) and force generation (**F4**, control: force = 8%, KCa: force = 20%). *P < 0.05.

Calcium-dependent potassium channel was dependent on intracellular calcium (Sah, 1996). KCa was involved in regulating the firing frequency of MNs (Powers and Binder, 2000). Previous experimental results showed a reduction of KCa in cat lumbar MNs during fictive locomotion (Brownstone et al., 1992). Modeling studies also suggested that reducing g_KCa_ increased MN excitability (Dai et al., 2002, 2018) and enhanced the recruitment of MN pools (Zhang and Dai, 2020). Similar to the modulation of VGSC, reducing g_KCa_ by 50% not only enhanced locomotor activities (Figures 4A,B) but also increased the frequency of MN firing (Figure 4C: from 33 to 55 Hz) and gain of MN output (Figure 4D: ΔK = 1.9 Hz/nA). Figure 4E illustrated the kinetics of the LTCC and KCa (m_LTCC_ and n_KCa_, Figure 4E1). The relationship between the tonic discharge and currents of KCa and LTCC was shown in Figure 4E2. Increasing the current of injection slightly decreased the amount of KCa currents in both control and g_KCa_ decrement, while LTCC currents barely changed (Figure 4E3). Reduction of g_KCa_ reduced the peak KCa current (Figure 4E3). The increased recruitment further facilitated locomotor patterns (Figure 4F1) and increased the ENG amplitude of extensor and flexor MN pools (by 28 and 26%, respectively, *P* < 0.05, Figure 4F2). The 50% reduction of g_KCa_ also dramatically facilitated the formation of locomotor patterns by increasing the locomotor vector (LV) from 0.83 to 0.97 and polar angle θ from 151° to 174° (Figure 4F3). More importantly, reducing g_AHP_ by 50% increased force production of extensor and flexor muscles by an average of 12% from 8 to 20% (Figure 4F4). These results implicated that modulation of g_KCa_ in MN pools could be a potential manner that motor system used to regulate locomotor activities and force generation during locomotion.

### Locomotor Activity Modulated by NaP of Motoneurons

**FIGURE 5:**
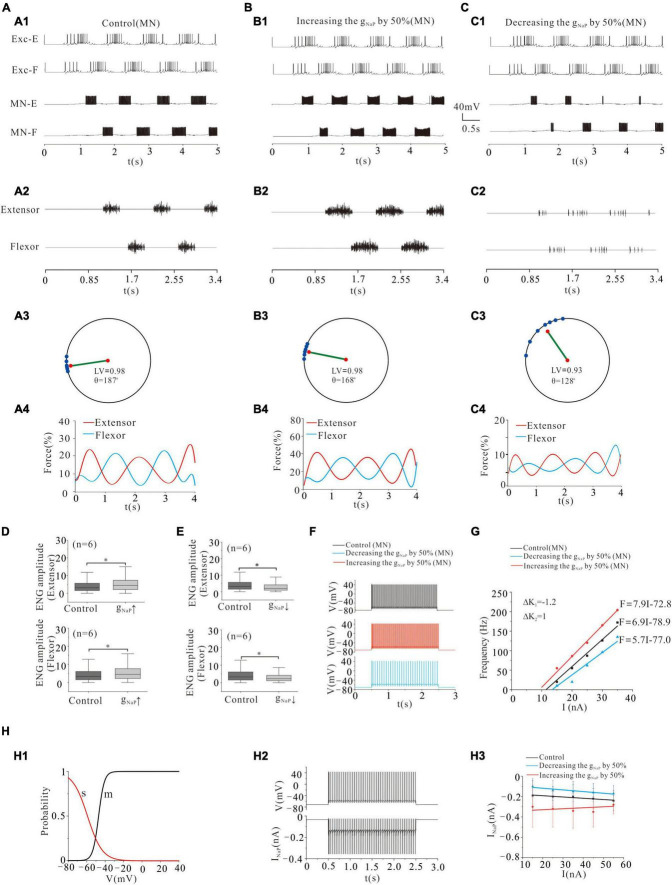
Locomotion regulated by NaP of MNs. **(A,B)**: Increasing g_NaP_ by 50% enhanced the locomotor activities **(A1,A2; B1,B2)**. The polar angle θ reduced from 187° to 168° **(A3,B3)** and force generation increased from 15 to 21% **(A4,B4)**. **(C)**: Reducing g_NaP_ by 50% reduced the locomotor activities **(C1,C2)**. The LV decreased from 0.98 to 0.93 **(C3)** and force generation from 15 to 6% **(C4)**. **(D)**: Increasing g_NaP_ by 50% increased ENG amplitude of extensor and flexor MN pools by 29 and 28%, respectively (*P* < 0.05). **(E)**: Contrarily, decreasing g_NaP_ by 50% decreased the ENG amplitude of extensor and flexor MN pools by 30 and 32%, respectively (*P* < 0.05). **(F)**: Repetitive firing was evoked by injecting a step current of 2-s duration and 16-nA into one of the selected MNs. A 50% increase of g_NaP_ increased the firing frequency of the MN (red curve, control: 33 Hz, g_NaP_: 50 Hz) while reducing g_NaP_ by 50% reduced the discharge rate of the MN (blue curve, control: 33 Hz, g_NaP_: 16 Hz). **(G)**: Increasing g_NaP_ by 50% shifted the F-I relationship to the left (red line), while a 50% reduction of g_NaP_ shifted the relationship to the right (blue line). **(H)**: The kinetics of NaP with activation (m) and slow inactivation curves (s) **(H1)**. The relationship between repetitive firing and peak NaP currents **(H2)**. The firing was evoked by injecting a 15.5 nA step current into the soma. Increasing injected currents increased the number of NaP currents in both control and g_NaP_ decrement, while slightly reducing NaP currents in g_NaP_ increment **(H3)**. *P < 0.05.

Persistent sodium conductance (g_NaP_) has been widely found in mammalian spinal neurons (Crill, 1996). Activation of g_NaP_ facilitates spike initiation and repetitive firing in rat and mouse spinal neurons (Lee and Heckman, 2001) and generates rhythmic activities during fictive locomotion (Tazerart et al., 2008; Brocard et al., 2013). In general, the function of NaP in MNs is different from that in INs, and the major distinction is that the NaP in MNs mediates persistent inward currents (PICs) and enables to generate repetitive firing in the presence of a sustained, depolarizing synaptic drive (Lee and Heckman, 2001; Harvey et al., 2006; Powers et al., 2012), while the NaP in INs contributes to locomotor oscillations, which rely on the NaP-dependent pacemaker properties (Brocard et al., 2013). In a recent modeling study, we demonstrated that enhancing NaP increased the excitability and output of spinal MNs (Dai et al., 2018; Zhang and Dai, 2020). In the present study, we further investigated the effect of modulating NaP of MN pools on locomotor patterns and force generation. Modeling results showed that increasing the conductance of NaP (g_NaP_) by 50% enhanced the formation of locomotor patterns (Figures 5A1,A2,B1,B2) and force generation of skeletal muscles (Figures 5A3,A4,B3,B4). The polar angle θ reduced from 187° to 168° (Figures 5A3,B3), and force generation increased from 15 to 21% (Figures 5A4,B4). And the ENG amplitude of extensor and flexor MN pools increased by 29 and 28%, respectively (Figure 5D: *P* < 0.05). On the contrary, however, reduction of g_NaP_ by 50% perturbed locomotor activities (Figures 5C1,C2) with reduction of LV from 0.98 (control) to 0.93, polar angle θ from 187° to 128° (Figure 5C3), and force generation from 15 to 6% (Figure 5C4). The ENG amplitude of extensor and flexor MN pools reduced by 30 and 32%, respectively (Figure 5E, *P* < 0.05). Figures 5F,G shows that increasing g_NaP_ by 50% increased the firing frequency of MN from 33 to 50 Hz (Figure 5F: red line) and enhanced the output of MN pools with a left-shift of the F-I relationship (Figure 5G: red line) while reducing g_NaP_ by 50% reduced the firing frequency from 33 to 16 Hz (Figure 5F: blue line) and decreased output of MN pools with a right-shift of the F-I relationship (Figure 5G: blue line). Figure 5H illustrates the kinetics of NaP with activation (m) and slow inactivation curves (s) (H1). Figure 5H2 shows the relationship between repetitive firing and peak NaP currents. The number of NaP currents increased with the increase of injected currents in both control and g_NaP_ decrement. However, the NaP currents decreased slightly with the increase of injected currents in g_NaP_ increment (H3). These results suggested that NaP channels play an essential role in regulating locomotor activities and force production during locomotion.

### Locomotor Activity Modulated by L-Type Calcium Channel of Motoneurons

**FIGURE 6:**
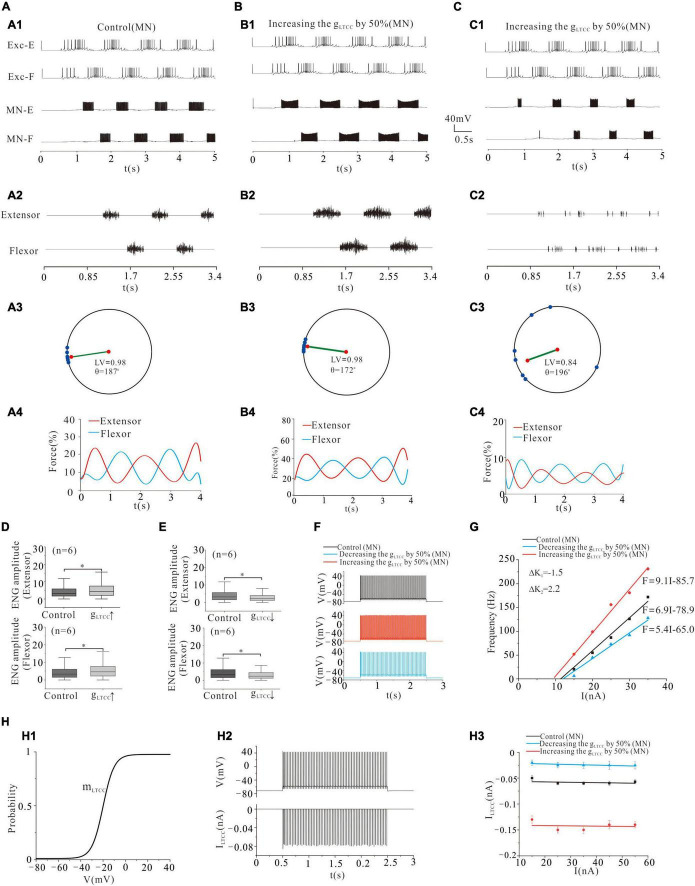
Locomotion regulated by LTCC of MNs. **(A,B)**: Increasing g_LTCC_ by 50% enhanced the rhythmic activity **(A1,A2; B1,B2)**. The polar angle θ reduced from 187° to 172° **(A3,B3)**, and the force generation increased from 15 to 30% **(A4,B4)**. **(C)**: Reducing g_LTCC_ by 50% reduced the rhythmic activity **(C1,C2)**. The LV decreased from 0.98 to 0.84 **(C3)** and force generation from 15 to 5% **(C4)**. **(D)**: Increasing g_LTCC_ by 50% increased ENG amplitude of both extensor and flexor MN pools by 32% (*P* < 0.05). **(E)**: Contrarily, decreasing g_LTCC_ by 50% reduced the ENG amplitude of extensor and flexor MN pools by 31 and 30%, respectively (*P* < 0.05). **(F)**: Repetitive firing was evoked by injecting a step current of 2-s duration and 16-nA into one of the selected MNs. A 50% increase of g_LTCC_ increased the firing frequency of the MN (red curve, control: 33 Hz, g_LTCC_: 63 Hz), while a 50% reduction of g_LTCC_ reduced the discharge rate of the MN (blue curve, control: 33 Hz, g_LTCC_: 26 Hz). **(G)**: Increasing g_LTCC_ by 50% increased slope of F-I relationship with left-ship of the F-I curve (ΔK_2_ = 2.2 Hz/nA, red line) while decreasing g_LTCC_ by 50% lowered slope of F-I relationship with right-ship of the F-I curve (ΔK_1_ = –1.5 Hz/nA, blue line). **(H)**: The kinetics of LTCC with activation (m_LTCC_) **(H1)**. The relationship between repetitive firing and peak LTCC currents **(H2)**. The firing was evoked by injecting 16.5 nA step current into the soma. The amount of LTCC currents barely changed with the increase of injected currents in both control and g_LTCC_ increment/decrement **(H3)**. *P < 0.05.

The LTCC was first reported in cat lumbar MNs in the 1980s (Schwindt and Crill, 1980a,b) and later in spinal INs (Hounsgaard and Kjaerulff, 1992; Dai and Jordan, 2011). In this study, we investigated the effect of modulating g_LTCC_ of MN pools on locomotor pattern formation and force generation. Similar to modulation of NaP, upregulation of g_LTCC_ by 50% facilitated the formation of locomotor patterns (Figures 6A1,A2,B1,B2) and force generation (Figures 6A1,A2,B3,B4). The polar angle θ reduced from 187° to 172° (Figures 6A3,B3) and force production increased from 15 to 30% (Figures 6A4,B4). Also, the ENG amplitude of extensor and flexor MN pools was increased by 32% (*P* < 0.05, Figure 6D). Contrarily, downregulation of g_LTCC_ by 50% disrupted the rhythmic activities and thus reduced the output of MN pools (Figures 6C1,C2). The LV decreased from 0.98 (control) to 0.84 (Figure 6C3) and force generation from 15 (control) to 5% (Figure 6C4). The ENG amplitudes of extensor and flexor MN pools reduced by 31 and 30% (*P* < 0.05, Figure 6E), respectively.

Similar to the results of NaP, Figures 6F,G show that a 50% increase of g_LTCC_ increased the frequency of MN firing from 33 to 63 Hz (Figure 6F: red line) and enhanced the output of MN pools with a left-shift of the F-I relationship (Figure 6G: red line), whereas a 50% reduction of g_LTCC_ reduced the firing frequency from 33 to 26 Hz (Figure 6F: blue line) and lowered output of MN pools with a right-shift of the F-I relationship (blue line, Figure 6G). Figure 6H illustrates the kinetics of LTCC with activation curve (m_LTCC_) (H1). Figure 6H2 shows the relationship between repetitive firing and peak LTCC currents. The amount of LTCC currents barely changed with the increase of injected currents in both control and g_LTCC_ increment/decrement (H3). In summary, simulation results suggested that the L-type Ca^2+^ currents played an important role in maintaining locomotor activities and force generation during locomotion.

### Effect of Channel Modulation of Central Pattern Generators Networks on the Generation of Locomotion

**FIGURE 7:**
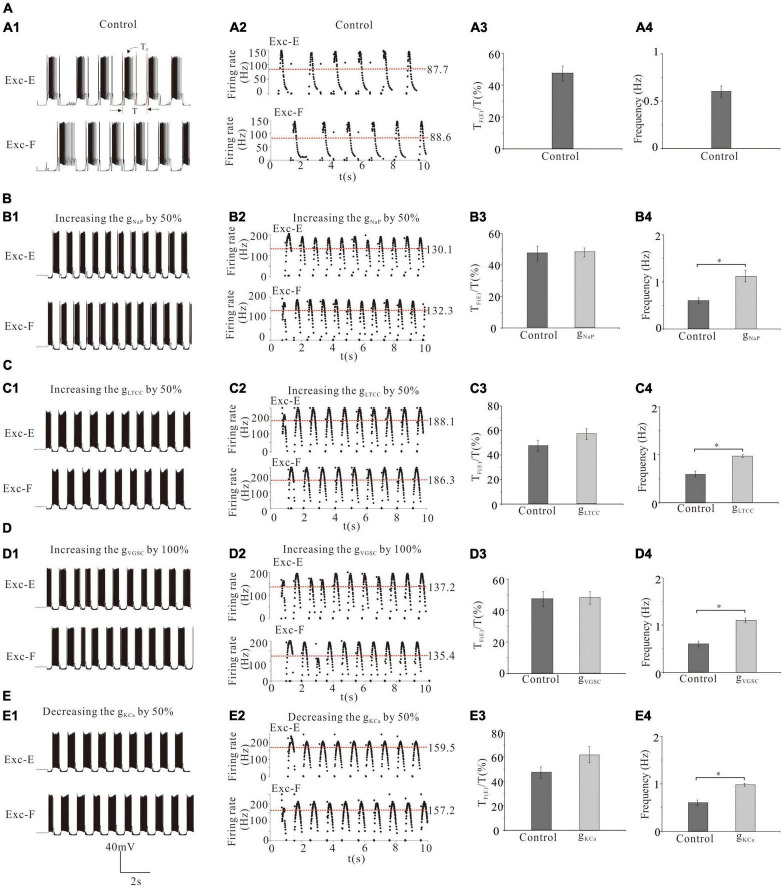
Effect of channel modulation of CPG networks on the generation of locomotion. **(A)**: The rhythm generator was mainly responsible for generating locomotor rhythm **(A1,A2)** from which the ratio of step cycle duration **(A3)** and frequency of step cycle **(A4)** were calculated. **(B)**: Increasing g_NaP_ by 50% increased the discharge rate of Exc-E and Exc-F from 87.7 and 88.6 Hz to 130.1 and 132.3 Hz **(B1,B2)**, the ratio of step cycle from 47 to 48% **(B3)**, and frequency of step cycle from 0.6 Hz to 1.12 Hz (**B4**, *P* < 0.05). **(C)**: Increasing g_LTCC_ by 50% increased the discharge rate of Exc-E and Exc-F from 87.7 and 88.6 Hz to 188.1 and 186.3 Hz, respectively **(C1,C2)**, the ratio of step cycle from 47 to 57% **(C3)**, and frequency of step cycle from 0.6 Hz to 0.97 Hz (**C4**, *P* < 0.05). **(D)**: Increasing g_VGSC_ by 100% increased the discharge rate of Exc-E and Exc-F from 87.7 to 88.6 Hz to 137.2 and 135.4 Hz, respectively **(D1,D2)**, the ratio of step cycle duration from 47 to 48% **(D3)**, and frequency of step cycle from 0.6 to 1.1 Hz (**D4**, *P* < 0.05). **(E)**: Decreasing g_KCa_ by 50% increased the discharge rate of Exc-E and Exc-F from 87.7 and 88.6 Hz to 159.5 and 157.2 Hz, respectively **(E1,E2)**, the ratio of step cycle from 47 to 62% **(E3)**, and frequency of step cycle from 0.6 Hz to 0.98 Hz (**E4**, *P* < 0.05). The red dash lines represent mean values of firing frequencies in **(A2,B2,C2,D2,E2)**. *P < 0.05.

In this above simulation, we demonstrated that channel modulation of MN pools altered MN recruitment and thus changed the ENG amplitude and force generation of flexor/extensor muscles. However, it remains unknown how to channel modulation of CPG networks, which could affect rhythmic generation during locomotion. Therefore, in this study, we investigated the effect of modulating channels in CPG networks on rhythmic generation and pattern formation. For the convenience of description, we defined the ratio of rhythmic step cycle (SC) by the formula: *SC = T_*F(E)*_/T*, where T_*E*_ and T_*F*_ were bursting duration of excitatory extensor and flexor INs, respectively, and T was a rhythmic period of step cycle (Figure 7A1).

Simulation results showed that a 50% increase of g_NaP_ increased the discharge rate of the rhythm generators (Exc-E and Exc-F) from 87.7 and 88.6 Hz (red dash line for mean value, control) to 130.1 Hz and 132.3 Hz (Figures 7A1,A2,B1,B2), the ratio of step cycle (T_*F*_/T or T_*E*_/T) from 47% (control) to 48% (Figures 7A3,B3), and frequency of step cycle from 0.6 Hz (control) to 1.12 Hz (Figures 7A4,B4, *P* < 0.05). Similar results were obtained for the modulation of other channels in rhythmic generators of the CPG. Simulation results showed that a 50% increase of g_LTCC_, a 100% increase of g_VGSC,_ or a 50% reduction of g_KCa_ significantly increased mean values of discharge rate of the Exc-E (Exc-F) pools by 100.4 (97.7), 49.5 (46.8), and 71.8 (68.6) Hz, respectively (Figures 7C1,C2,D1,D2,E1,E2, red dash line for mean value). The same channel modulations increased the ratio of step cycle by 10, 1, and 15% (Figures 7C3,D3,E3) and frequency of step cycle by 0.37, 0.5, and 0.38 Hz (Figures 7C4,D4,E4, *P* < 0.05), respectively. The above results suggested that upregulation of g_LTCC_, g_NaP_, and g_VGSC_ or downregulation of g_KCa_ in rhythm generators of the CPG facilitated rhythmic generation and pattern formation during locomotion.

### Locomotor Activity Modulated by NaP of Rhythmic Generator

**FIGURE 8:**
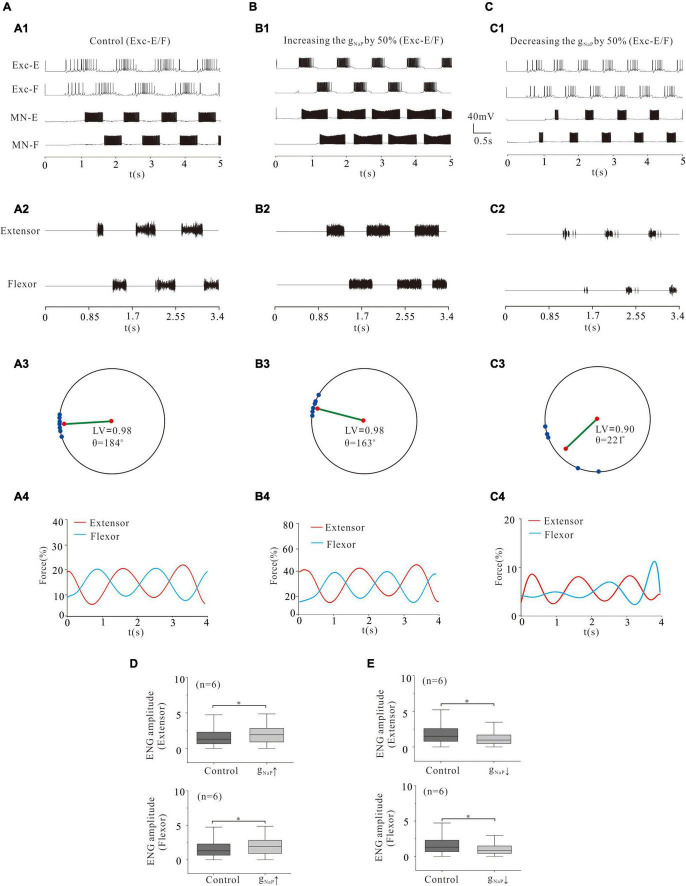
Locomotion regulated by NaP of rhythm generators. **(A,B)**: Increasing g_NaP_ by 50% enhanced the locomotor activities **(A1,A2; B1,B2)**. The polar angle θ reduced from 184° to 163° **(A3,B3)** and force generation increased from 15 to 25% **(A4,B4)**. ENG amplitude of extensor and flexor MN pools increased by 25 and 20% (*P* < 0.05, **D**), respectively. **(C)**: Reducing g_NaP_ by 50% reduced locomotor activities in rhythm generators **(C1,C2)**. The LV decreased from 0.98 to 0.9 **(C3)** and force generation decreased from 15 to 5% **(C4)**. Furthermore, the ENG amplitude of flexor and extensor MN pools decreased by 35 and 37% (*P* < 0.05, **E**), respectively. *P < 0.05.

In the above simulation, we demonstrated that modulation of ionic channels in MN pools indeed affected rhythmic activities of locomotion and force production of skeletal muscles. In the following simulation, we further explored the effect of the same channel modulations in the CPG networks (rhythmic generator) on the locomotor pattern formation and force generation. Similarly, we started with NaP. A 50% increase of g_NaP_ in rhythmic generator (Exc-E and Exc-F pools) enhanced the rhythmic bursting (Figures 8A1,B1) and ENG activity (Figures 8A2,B2). The polar angle θ reduced from 184° to 163°, and force generation increased from 15% (control) to 25% (Figures 8A4,B4). The ENG amplitude of extensor and flexor MN pools increased by 25 and 20%, respectively (*P* < 0.05, Figure 8D). On the other hand, however, a 50% reduction of g_NaP_ significantly reduced rhythmic bursting (Figures 8A1,C1) and locomotor activities (Figures 8A2,C2). The LV decreased from 0.98 (control) to 0.9 (Figures 8A3,C3), and the force generation from 15% (control) to 5% (Figures 8A4,C4). The ENG amplitudes of extensor and flexor MN pools decreased by 35 and 37% (Figure 8E, *P* < 0.05), respectively. These simulation results implicated that modulation of NaP in CPG networks altered the locomotor pattern formation and force production.

### Locomotor Activity Modulated by L-Type Calcium Channel of Rhythmic Generator

**FIGURE 9:**
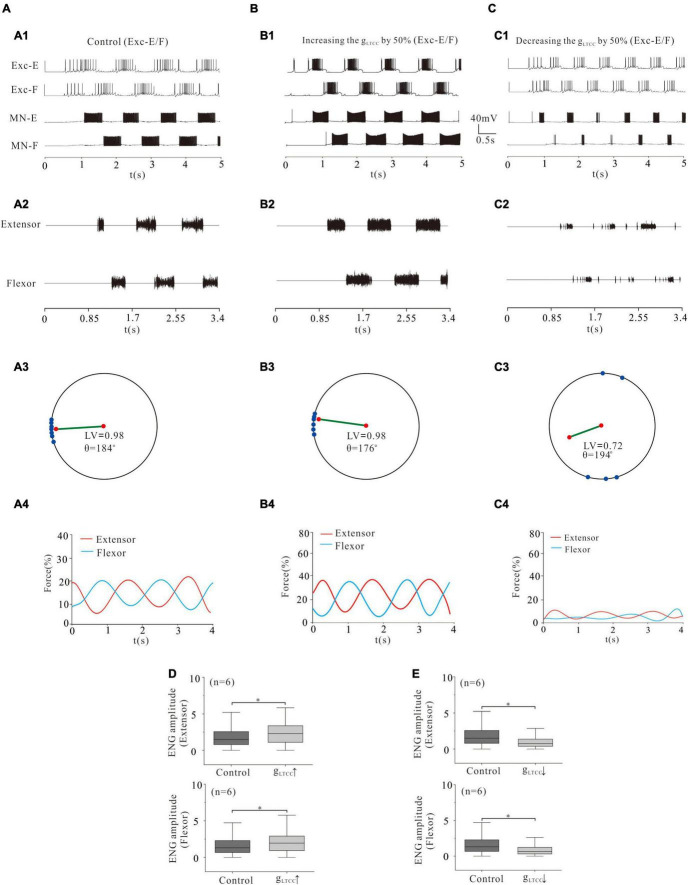
Locomotion regulated by LTCC of rhythm generators. **(A,B)**: Increasing g_LTCC_ by 50% enhanced the locomotor activities **(A1,A2; B1,B2)** with a reduction of θ from 184° to 176° **(A3, B3)**. The force generation increased from 15 to 21% **(A4,B4)**. ENG amplitude of extensor and flexor MN pools increased by 28% and 25% (*P* < 0.05, **D**), respectively. **(C)**: However, reducing g_LTCC_ by 50% reduced locomotor output **(C1,C2)**. The LV decreased from 0.98 to 0.72 **(C3)** and the force generation decreased from 15 to 5% **(C4)**. The ENG amplitude of extensor and flexor MN pools decreased by 50 and 49% (*P* < 0.05, **E**), respectively. *P < 0.05.

Similar to the simulation of NaP modulation, increasing g_LTCC_ significantly enhanced rhythmic activities (Figures 9A1,A2,B1,B2) and force generation (Figures 9B3,B4). Specifically, a 50% increase of g_LTCC_ reduced the polar angle θ from 184° to 176° and increased force production from 15 to 21% (Figures 9A3,A4,B3,B4). The ENG amplitude of extensor and flexor MN pools increased by 28 and 25%, respectively (Figure 9D, *P* < 0.05). On the contrary, however, a 50% reduction of g_LTCC_ almost removed locomotor activities (Figures 9A1,A2,C1,C2). The LV reduced from 0.98 (control) to 0.72 and the force production from 15 to 5% (Figures 9A3,A4,C3,C4). The ENG amplitude of extensor and flexor MN pools reduced by 50 and 49%, respectively (Figure 9E, *P* < 0.05). These results suggested that modulation of L-type Ca^2+^ channels in CPG networks regulated the rhythmic generation of locomotion and force production of skeletal muscles. Furthermore, modulation of LTCC and/or NaP in the CPG networks determined pattern formation of locomotion, coordination of limb movement, and force production of muscles.

### Locomotor Activity Modulated by L-Type Calcium Channel and NaP of Central Pattern Generators Networks and MNs

**FIGURE 10:**
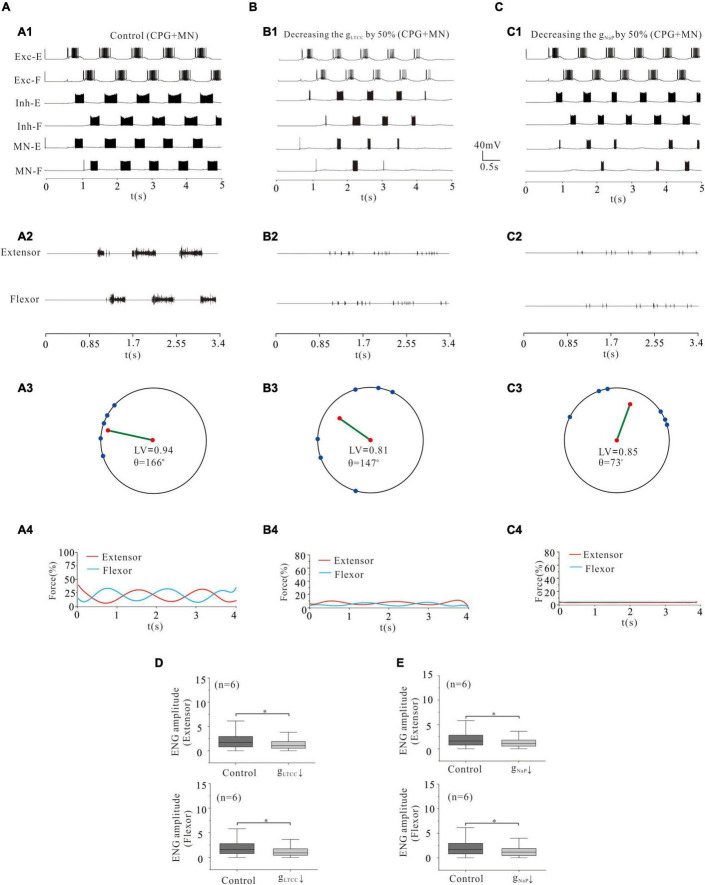
Locomotion regulated by LTCC and NaP of CPG networks and MNs. **(A,B)**: Reduction of g_LTCC_ by 50% in both CPG networks (Exc-E/F and Inh-E/F) and MN pools reduced the locomotor activity **(A1,A2; B1,B2)**. The LV decreased from 0.94 to 0.81 **(A3,B3)** and force generation from 25 to 5% **(A4,B4)**. ENG amplitude of extensor and flexor MN pools was reduced by 39 and 41%, respectively (*P* < 0.05, **D**). **(C)**: Decreasing g_NaP_ by 50% reduced locomotor output **(C1,C2)**. The LV decreased from 0.94 to 0.85 **(C3)** and the force generation from 25 to 2% **(C4)**. The ENG amplitude of extensor and flexor MN pools was reduced by 33 and 32%, respectively (*P* < 0.05, **E**). *P < 0.05.

In order to further investigate the modulation of ionic channels for locomotor activity, we simultaneously reduced the conductance of LTCC or NaP channels in rhythmic generator (Exc-E and Exc-F), inhibitory INs (Inh-E and Inh-F), and MNs (MN-E and MN-F). Simulation results showed that a 50% reduction of g_LTCC_ in the CPG and MN networks dramatically reduced the rhythmic bursting in the whole networks (Figures 10A1,B1) and locomotor activity (Figures 10A2,B2). The locomotor pattern was disrupted completely with the LV reduced from 0.94 (control) to 0.81 (Figures 10A3,B3) and the force generation reduced from 25% (control) to 5% (Figures 10A4,B4). The ENG amplitude of extensor and flexor MN pools reduced by 39 and 41%, respectively (Figure 10D, *P* < 0.05). Similarly, a 50% reduction of g_NaP_ in the CPG and MN networks significantly reduced rhythmic bursting (Figures 10A1,C1) and locomotor activity (Figures 10A2,C2). The locomotor pattern was almost removed completely with the LV decreased from 0.94 (control) to 0.85 (Figures 10A3,C3) and the force generation from 25% (control) to 2% (Figures 10A4,C4). The ENG amplitude of extensor and flexor MN pools was reduced by 33 and 32%, respectively (Figure 10E, *P* < 0.05). We further reduced the conductance of LTCC or NaP by 80% in the CPG and MN networks, and the locomotor activity was completely inhibited from the networks (not shown). These results suggest that modulation of LTCC or NaP channels in whole spinal networks altered the locomotor activity, ENG amplitude, and force generation.

### Pharmacological Investigation of Fictive Locomotion Modulated by L-Type Calcium Channel and NaP

The above results predict some potential channel mechanisms underlying the generation of locomotion. In this study, we used blind patch-clamp and ENG recording techniques to investigate the effects of modulating LTCC and NaP channels on the F-I relationships of lumbar MNs and fictive locomotion in isolated spinal cord preparations of neonatal SD rats (postnatal days 1–4).

**FIGURE 11:**
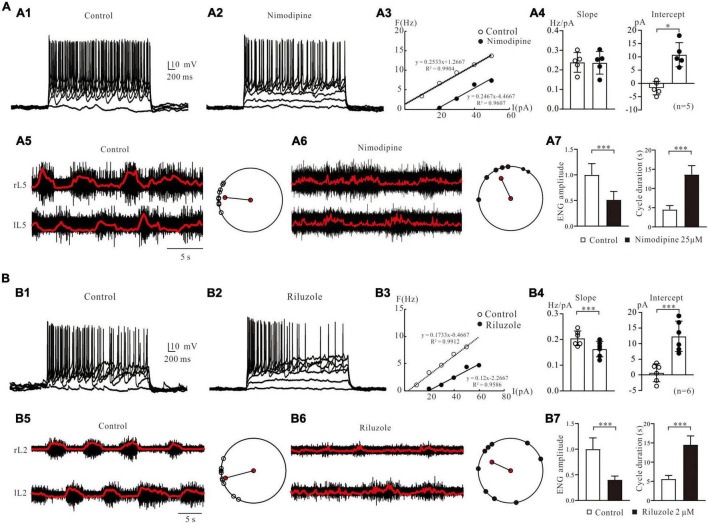
Pharmacological investigation of fictive locomotion modulated by LTCC and NaP. **(A)**: F-I relationships and fictive locomotion regulated by L-type calcium channels. **(A1)**: Repetitive firing was evoked by injecting step currents of 3-s duration and 10-pA step into a spinal MN of a neonatal rat spinal cord. **(A2)**: Bath application of 25 μM nimodipine reduction MNal excitability. **(A3)**: F-I relationships were calculated in control and nimodipine. **(A4)**: Statistical results (slopes and intercepts of F-I relationship) from 5 MNs were averaged, and nimodipine decreased the slopes (*P* > 0.05, **A4**, left) with significantly shifted the F-I relationships to the right (ΔI = 12.6 ± 6 pA, *P* < 0.05, **A4**, right). **(A5)**: Electroneurogram recordings (right panel) of fictive locomotion from the right and left of L5 were induced by bath application of 5-HT (15 μM) and NMDA (3 μM). The polar plots (right panel) showed a well-coordinated activity. **(A6)**: Application of nimodipine (25 μM) disrupted locomotor activity. Polar plots showed the disorganized rhythmic activity. **(A7)**: Summary diagrams showed the ENG amplitude (left) and cycle duration (right) recorded in control and in the presence of nimodipine. **(B)**: F-I relationships and fictive locomotion regulated by persistent sodium channels. **(B1)**: Repetitive firing was elicited by injecting the step currents into another spinal MN. **(B2)**: Bath administration of 2 μM riluzole decreased motoneuronal excitability **(B3)**: F-I relationships were calculated in control and riluzole. **(B4)**: Statistical results from 6 MNs showed that riluzole decreased the slopes (ΔK = –0.04 ± 0.01 Hz/pA, *P* < 0.001, left) with significantly shifted the F-I relationships to the right (ΔI = 12 ± 2.5 pA, *P* < 0.001, right). **(B5)**: ENG (left) of fictive locomotion activity from the right and left of L2 were induced by 5-HT (15 μM) and NMDA (3 μM) and the polar plots (right) showed well-coordinated activity. **(B6)**: Application of riluzole (2 μM) disrupted locomotor activity. Polar plots showed the disorganized rhythmic activity. **(B7)**: Summary diagrams showed the ENG amplitude (left) and cycle duration (right) recorded in control and in the presence of riluzole. Error bars show SD. *P < 0.05, ^***^P < 0.001.

Bath application of 25 μM nimodipine, an antagonist of LTCCs (Cav1.3), decreased MNs excitability (Figures 11A1,A2). The F-I relationship of this MN was established through injection of a series of step current pulses (Figure 11A3). Nimodipine shifted the F-I relationship to the right (ΔI = 23.1 pA; control: I-intercept = −5 pA, nimodipine: I-intercept = 18.1 pA) with little change in the slope (ΔK = −0.007 Hz/pA; control: K = 0.2533 Hz/pA, nimodipine: K = 0.2467 Hz/pA). Statistical results showed that nimodipine significantly shifted the F-I relationships to the right (Figure 11A4, right panel: control: I-intercept = −1.8 ± 2.2 pA, nimodipine: I-intercept = 10.8 ± 4.1 pA, ΔI = 12.6 ± 6 pA, *P* < 0.05, *n* = 5) without substantial alteration of the slope (Figure 11A4, left panel: control: K = 0.24 ± 0.05 Hz/pA, nimodipine: K = 0.24 ± 0.05 Hz/pA, ΔK = −0.005 ± 0.006 Hz/pA, *P* = 0.2, *n* = 5). These data suggest that nimodipine decreased lumbar motoneuronal excitability and thus firing rate by shifting the F-I relationship to the right.

In the following study, we further examined the effect of LTCCs on the generation of fictive locomotion. 5-HT (15 μM) and NMDA (5 μM) were applied to the recording bath to generate well-coordinated fictive locomotion, including alternation between the right and left of L5 (Figure 11A5). The raw ENG recordings of the right (R) and left (L) L5 ventral roots were recorded over a 25-s duration (Figure 11A5). The ENG data were then rectified and filtered for detailed analysis to determine the relationships between right/left activities (Figure 11A5, red line). Polar plots (Figure 11A5 right), which were produced from the rectified and filtered waveforms (Figure 11A5, right panel), demonstrated well-coordinated locomotor activities. The right and left ENGs (r L5 vs. l L5) were out of phase (θ = 174°, LV = 0.85), indicating right/left alternation. A 2 μM nimodipine perturbed the locomotor activities and decreased the amplitudes and frequency of the ENG bursts (Figure 11A6, left). As shown in Figure 11A6, left panel, the left-right coordination was disrupted after 5 min of nimodipine administration (θ = 105°, LV = 0.71). We analyzed the effect of nimodipine on ENG amplitude and cycle duration. Nimodipine significantly reduced the ENG amplitude by 49% (Figure 11A7, left, *P* < 0.001) and extended cycle duration by 9.4 s (control: 4.3 ± 0.6; nimodipine: 13.7 ± 2.5 s, *P* < 0.001, Figure 11A7, right), which was calculated as the time difference between the onset of two consecutive ENG bursts.

The contribution of NaP channels to MN excitability and fictive locomotion was also specifically explored in this study. The repetitive firing was recorded from a lumbar MN of a P3-rat spinal cord (Figure 11B1). Bath application of 2 μM riluzole reduced repetitive discharge of the MN (Figure 11B2) and shifted the F-I relationship to the right (ΔI = 16.2 pA; control: I-intercept = 2.7 pA, riluzole: I-intercept = 18.9 pA) with reduction of the slope (ΔK = −0.0533 Hz/pA; control: K = 0.1733 Hz/pA, riluzole: K = 0.12 Hz/pA, Figure 11B3). Statistical results from 6 MNs showed that riluzole significantly induced a right-shift of F-I relationship (12 ± 2.5 pA, control: 0.3 ± 2.7 pA, riluzole: 12.4 ± 4.4 pA, *P* < 0.001, Figure 11B4, right panel) and decreased the slope from 0.21 ± 0.02 Hz/pA to 0.16 ± 0.03 Hz/pA (ΔK = −0.04 ± 0.01 Hz/pA, *P* < 0.001, Figure 11B4, left panel). A 2 μM riluzole also blocked fictive locomotion in the rat spinal cord. A typical example was shown in Figure 11 (B5 and B6), where well-coordinated locomotion was induced in L2 of an isolated spinal cord through bath application of 15 μM 5-HT and 5 μM NMDA (θ = 192°, LV = 0.95, Figure 11B5, right). After 2 μM riluzole application an impairment of left-right coordination was observed (θ = 147°, LV = 0.56, Figure 11B6, right). The locomotion was largely slowed down and almost completely removed. Statistical analysis indicated that riluzole dramatically reduced the ENG amplitude by 61% (*n* = 6, *P* < 0.001, Figure 11B7, left) and prolonged cycle duration by 8.9 s (control: 5.7 ± 0.5 s; riluzole: 14.6 ± 2.4 s, *P* < 0.001, Figure 11B7, right). These experimental results suggested that LTCC and NaP channels play an essential role in modulating locomotion in the neonatal rat spinal cord, consistent with the simulation results (Figure 10).

We noted that the F-I relationship with blockade of NaP by riluzole or LTCC by nimodipine was different from that of simulation data in terms of changes in the slope and amount of right-shifting of the F-I curve (Figure 5G vs. Figure 11B3 or Figure 6G vs. Figure 11A3). We believed that this difference was due to the different membrane properties of the spinal MNs between the cat (modeling) and rat (experiment).

## Discussion

Using modeling and electrophysiological approaches, we demonstrated that modulation of ionic channels in spinal MNs as well as CPG networks changed neuronal excitability and MN recruitment and thus altered rhythmic generation of locomotion and force production of flexor-extensor muscles. Simulation results suggested that upregulation of g_VGSC_, g_NaP_, or g_LTCC,_ or downregulation of g_KCa_ significantly enhanced locomotor rhythm and force generation during locomotion. In addition, modulation of LTCCs or NaP channels in whole spinal networks altered the locomotor activity, ENG amplitude, and force generation. The physiological experiment results from the isolated spinal cord of neonatal rats supported the simulation predictions that NaP and LTCC played an essential role in generating locomotion.

### Modeling of Central Pattern Generators Networks for Rhythmic Generation and Locomotor Activities

Numerous CPG models with multiple levels have been developed to interpret the diversity of rhythmic generation and locomotor activities in vertebrates. The two-level of CPG model finely described how sensory stimulation altered locomotor patterns (Kriellaars et al., 1994), and the three-level CPG model well explained the changes of rhythmic output during fictive locomotion (Rybak et al., 2006a,b; McCrea and Rybak, 2008). A more complicated model of spinal circuits with four rhythmic generators and inhibitory commissural and fore-hind inhibitory interactions unveiled the mechanism underlying the speed-dependent expression of different gaits between different limbs and interlimb coordination (Danner et al., 2016, 2017). These CPG models demonstrated the importance of the structure of neural networks that play a major role in generating locomotion. However, it is still unclear how to channel modulation in both CPG networks and MN pools that could affect locomotor activity. In order to explore this issue, we built a highly simplified two-level CPG model which was capable of generating locomotor rhythm and pattern. More importantly, some major conductances which are widely found in spinal neurons are included in the model. This model not only duplicated locomotor activities as observed in physiological experiments but also demonstrated that modulation of NaP, VGSC, LTCC, and/or KCa altered the locomotor pattern and force generation. The simulation prediction about the roles of NaP and LTCC in generating locomotion was verified by electrophysiological experiments (Figures 10, 11). In order to simplify the simulation process in this study, only the FF-type MNs were integrated into the present model. However, the general conclusions drawn from the present simulations are applicable to the S- and FR-type MN models as shown in our previous studies (Dai et al., 2018, 2002; Zhang and Dai, 2020). Another important approach we used to convert the simulated ENG bursting to force production of skeletal muscles was the mathematical modeling of neuromusculoskeletal interaction. In fact, in order to quantify the relationship between the recorded ENG signals of MN pools and generated force of innervated skeletal muscles, several approaches have been developed (Teka et al., 2017; Dideriksen et al., 2019). Especially, muscle contractility has been well described as the neuromusculoskeletal interaction in the previous studies (Prilutsky and Zatsiorsky, 2002). In this study, we quantified the force generation of CPG networks based on the models from these studies. However, we focused only on the force generation by motoneuronal signals (ENG) without considering the detailed influence of musculoskeletal activity on locomotion. We actually input the ENG signals into the musculoskeletal model to quantitatively convert the ENG bursting to the strength of the muscle force. The simulation results were compared to experimental observation. Finally, the present CPG model could be further developed as multi-limbs CPG models to study the channel mechanisms underlying the generation of locomotion and coordination of limbs during walking.

### Modulation of L-Type Calcium Channel and NaP in the Central Pattern Generators and MN Networks

In addition to separately studying channel modulation in CPG networks and MN pools, we also examine the integration of channel modulating in both the CPG and MN networks. Since the purpose of this study is to explore channel mechanisms underlying locomotion and force generation, therefore we focused not only on the MNs (Figures 3–6) but also on the excitatory component (Exc-E/F) of the CPG networks (Figures 7–9), which have been proposed to play an essential role in the rhythmic generation and pattern formation during locomotion (McCrea and Rybak, 2008). Furthermore, we further investigated the modulation of LTCC or NaP channels in both CPG networks (Exc-E/F and Inh-E/F) and MN pools to explore the effect of LTCC or NaP on locomotion (Figure 10). The simulation results showed that blockage of LTCC or NaP in whole spinal circuits inhibited the rhythmic generation of the networks and removed force production of the skeletal muscle. These results were consistent with the isolated spinal cord experiment (Figure 11) and previous studies (Büschges et al., 2000; Zhong et al., 2007; Tazerart et al., 2008; Brocard et al., 2010). It would be significant to further investigate the integrated contributions from other channels to the generation of locomotion and force production of skeletal muscles in the future study.

### Locomotion Modulated by Channels From Central Pattern Generators to Force Generation

The novel point of this study was to systematically study the generation of locomotion modulated by ionic channels from neuronal excitability to rhythmic generation, pattern formation, MN recruitment, ENG signals, and force production. The neuronal excitability was mainly described by the F-I relationship. Increasing g_VGSC_, g_NaP_, and g_LTCC_ or decreasing g_KCa_ in MN pools significantly increased the gain of the F-I relationship, accompanied by an increase in firing frequency of the MNs (Dai et al., 2018). Furthermore, these channel modulations induced non-linear facilitation of recruitment of MN pools (Zhang and Dai, 2020), increment of ENG amplitudes, and enhancement of force generation for locomotion. However, from the perspective of CPG networks, the same channel modulations increased discharge rates of CPG networks (rhythmic generators), facilitated rhythmic generation and pattern formation. These simulation results suggested that ionic channels could be widely involved in the modulation of CPG networks and MNs, thus regulating locomotion and force production.

### Step Cycle and Period Modulated by Channels

One of the major characteristics of CPG networks is their hierarchical structures with each level being of specific functions (McCrea and Rybak, 2008). Rhythmic generators (Exc-E and Exc-F) have been proposed in CPG networks for rhythmic generation and gait coordination (McCrea and Rybak, 2007). Our simulation supported these roles of rhythmic generator in initiating locomotion. Moreover, we showed that the duration of step cycles and frequency of the period of the cycles could be substantially regulated by modulating VGSC, NaP, LTCC, and/or KCa in the rhythmic generator (Figure 7). These results appeared to be functionally different from those induced by the same channel modulation in MN pools, suggesting that the channel mechanisms could produce different effects on locomotion, depending on what types of neuron populations are being modulated.

### Modeling Prediction and Whole Spinal Cord Experiments

In this study, we did whole rat spinal cord experiments to examine some of the predictions from our simulations of cat spinal cord. Comparing with the previous studies of intrinsic membrane properties of spinal MNs in cats (Hochman and McCrea, 1994; Krawitz et al., 2001) and rats (Xie and Ziskind-Conhaim, 1995; Krutki et al., 2017), we find that difference in the properties between these two species change with different parameters. Small difference include a 17% difference in resting membrane potential (cat: −66.8 ± 6.2; rat: −62.39 ± 8.7 mV), 1% in AP threshold (−44.1 ± 9.3; −43.6 ± 1.5 mV), 17% in AP amplitude (58.5 ± 9.7; 68.28 ± 9.97 mV), and 32% in rheobase (11.6 ± 3.1; 7.89 ± 4.66 nA), whereas big difference is observed in AP half-width (cat: 2.2 ± 0.4; rat: 0.55 ± 0.09 ms; 75%), AHP half decay (81.9 ± 17.1; 16.43 ± 6.87 ms; 80%) and input resistance (.91 ± 0.2; 2.42 ± 1.16 MΩ; 168%). Since AP threshold, AP amplitude, AHP amplitude, and rheobase are essential parameters dominating the neuronal active membrane properties, the substantial similarities of these parameters between cat and rat allow us to use experimental results from the whole rat spinal cord to verify the simulation results from modeling of cat spinal cord.

The experimental data confirmed the simulation predictions that reduction or blockade of LTCCs or NaP current reduced MN output and ENG amplitude and completely disrupted locomotor activities. In exploring the ionic basis for rhythmogenesis, previous studies identified the NaP current as a critical current in the burst-generating mechanism. Inhibition of NaP abolishes locomotor-like activity in rodents (Zhong et al., 2007; Brocard et al., 2010) and salamanders (Ryczko et al., 2010) and disrupts locomotion in zebrafish (Song et al., 2020) and Xenopus laevis tadpoles (Svensson et al., 2017). In recent modeling studies, we found that NaP plays an important role in MN output and recruitment (Dai et al., 2018; Zhang and Dai, 2020). Blockade of L-type channels by nimodipine also decreased the frequency and increased the duration of the locomotor bursts (Büschges et al., 2000). The simulation results of the present study are basically consistent with the previous studies. The effects of blocking NaP and LTCC channels on ENG recordings were much similar to the simulation results of downregulating g_NaP_ and g_LTCC_ in the whole spinal network (Figures 10, 11), in terms of reducing the ENG amplitude and disrupting the rhythmic activity. It was shown in previous genetic studies that reducing the levels of the voltage-gated calcium channel in MNs led to defective locomotion in Drosophila larval (Chang et al., 2014), consisting with the prediction of the present simulation. Although in whole spinal cord experiments we focused on the effect of nimodipine and riluzole on the alternation of the left and right limbs, which was different from the alternation of flexors and extensors in the model, the key point we showed these data was to verify that nimodipine and riluzole perturbed the fictive locomotion in isolated spinal cord no matter it was observed in the rhythmic activities of contralateral pair of L2 or L5 or ipsilateral pair of L2 and L5. Both modeling and experiment results suggested that NaP and LTCC are two key channels participating in generating locomotion.

## Conclusion

Using modeling and experimental approaches we investigated channel mechanisms regulating locomotor patterns and force generation in the cat spinal cord during locomotion. Simulation results demonstrated that upregulation of VGSC, NaP, or LTCC or downregulation of KCa channels in either MN pools or CPG networks increased the MN output and recruitment, facilitated the generation of locomotion, increased ENG amplitude, and enhanced force generation of flexor-extensor muscles. In particular, NaP and LTCC channels played an essential role in initiating and regulating locomotion.

## Data Availability Statement

The original contributions presented in the study are included in the article/supplementary material, further inquiries can be directed to the corresponding author/s.

## Ethics Statement

The animal study was reviewed and approved by Animal Experiment Ethics Committee of East China Normal University.

## Author Contributions

YD proposed and conducted the research plan. QZ designed and performed simulation. YC performed electrophysiological experiments. QZ and YC drafted the manuscript. YD, QZ, and YC revised the manuscript. MZ participated in modeling setting and manuscript revision. YD, QZ, YC, and MZ approved the final version of the manuscript. All authors contributed to the article and approved the submitted version.

## Conflict of Interest

The authors declare that the research was conducted in the absence of any commercial or financial relationships that could be construed as a potential conflict of interest.

## Publisher’s Note

All claims expressed in this article are solely those of the authors and do not necessarily represent those of their affiliated organizations, or those of the publisher, the editors and the reviewers. Any product that may be evaluated in this article, or claim that may be made by its manufacturer, is not guaranteed or endorsed by the publisher.
